# Metabolomic Analysis of Key Metabolites and Regulatory Mechanisms in the Transition of Uterine Receptivity in Water Buffalo (*Bubalus bubalis*)

**DOI:** 10.3390/metabo15090615

**Published:** 2025-09-17

**Authors:** Xingrong Lu, Jingyuan Song, Gan Liang, Huapei Zhong, Yuanyuan Xu, Yingxue Xie, Deshun Shi, Chan Luo

**Affiliations:** 1 Guangxi Key Laboratory of Animal Breeding, Disease Control and Prevention, College of Animal Science and Technology, Guangxi University, 75 Xiuling Road, Nanning 530005, China; luxingrong074@163.com (X.L.); 2218301022@st.gxu.edu.cn (J.S.); 2 Guangxi Key Laboratory of Buffalo Genetics, Reproduction and Breeding, Guangxi Buffalo Research Institute, Chinese Academy of Agricultural Science, Nanning 530001, China; 18776452585@163.com (G.L.); 15878178301@163.com (H.Z.); xuyyswu@163.com (Y.X.); woshixuanyue@163.com (Y.X.)

**Keywords:** water buffalo, early pregnancy, serum metabolomics, endometrial receptivity, immune remodeling

## Abstract

**Background:** While economically vital, buffalo exhibits low reproductive efficiency largely due to embryonic losses during implantation. Successful implantation requires precise embryo–maternal communication and metabolic/immune adaptations in the endometrium. We aimed to identify key serum metabolic signatures and associated peripheral immune responses that characterize the endometrial receptivity window during early pregnancy in water buffalo. **Methods:** Blood samples from pregnant (Preg, *n* = 12) and non-pregnant (Non-P, *n* = 10) buffaloes were collected on days 15, 18, and 21 post-artificial insemination (AI). We measured leukocyte counts and hormone levels and performed untargeted serum metabolomic profiling using LC-MS. **Results:** Pregnant buffaloes showed significantly reduced total white blood cell count, lymphocyte (LYM%), and neutrophil (NEU%) percentages, indicating immune remodeling at the beginning of pregnancy establishment. Metabolomic analysis identified 131 differentially expressed metabolites (DEMs) associated with pregnancy status at different stages. Enriched pathways included steroid hormone synthesis, retinol metabolism, starch/sucrose metabolism, and phenylalanine biosynthesis. Crucially, alterations in unsaturated fatty acids, retinol, and phenylalanine metabolism, along with monocyte (MON%)/LYM% ratios, were strongly linked to receptivity changes and successful implantation. **Conclusions:** Endometrial receptivity in buffalo during the embryonic implantation window was associated with changes in immune cells and metabolism in the blood, suggesting that immunometabolism may play an essential role in modulating endometrial receptivity during the implantation window. This study provides potential clues and a metabolic framework for understanding the underlying mechanisms of buffalo embryonic implantation.

## 1. Introduction

The embryonic implantation window—a restricted temporal phase characterized by endometrial receptivity—constitutes a pivotal developmental stage for successful pregnancy in mammalian species [1]. This temporally constrained interval, defined by uterine receptivity and developmental synchronization between the embryo and uterus, is fundamental to embryo adhesion, invasion, and subsequent placentation [2,3,4]. Dysregulation of this exquisitely orchestrated process represents a major contributor to early pregnancy loss and diminished reproductive efficiency [5,6]. While the existence and biological significance of this window are well-established, the intricate molecular mechanisms governing its precise spatiotemporal regulation—particularly the dynamic metabolic interactions mediating uterine–embryonic crosstalk—remain incompletely elucidated.

Metabolomics constitutes a robust analytical tool for decoding these complex biological processes [7]. The metabolome functions as a direct functional readout of cellular activity and physiological state [8]. Alterations in circulating metabolites mirror perturbations in energy metabolism, signaling cascades, and substrate availability, which are hypothesized to modulate the microenvironment to facilitate embryonic–endometrial communication and adhesion [9]. Serum metabolites, which integrate systemic and local endometrial metabolic activities, are poised to act as key regulators of critical processes during this period, including immunomodulation, cellular proliferation, vascular remodeling, and signal transduction [9,10,11]. Accumulated evidence indicates that perturbations in specific metabolic pathways (e.g., those involving amino acids, lipids, and energy substrates) exert profound effects on endometrial receptivity, trophoblast function, and implantation success [12,13,14]. Furthermore, shifts in metabolic profiles correlate with altered expression of receptivity markers and adhesion molecules, rendering them valuable indicators of reproductive status and potential predictive biomarkers for pregnancy outcomes [15,16,17].

Buffalo (Bubalus bubalis) possesses substantial global economic significance, particularly in tropical regions. However, relative to cattle, buffalo exhibit inherently lower reproductive efficiency, posing a significant constraint on their productivity [18]. Enhancing reproductive efficiency, particularly via advanced biotechnologies such as embryo transfer (ET), is therefore imperative for optimizing productivity in this species [19,20]. A critical bottleneck limiting the success of ET programs lies in determining the optimal timing for embryo transfer into recipient animals to achieve precise alignment with their respective implantation windows—a synchrony essential for maximizing pregnancy rates [20,21,22,23]. Accurately defining the temporal dynamics and metabolic drivers of the implantation window is thus paramount for improving pregnancy outcomes. In buffalo, Days 15, 18, and 21 post-insemination may correspond to the critical period of endometrial transformation [24,25]. During this time, pregnancy-associated glycoprotein (PAG), estradiol (E2), and progesterone (P4) may undergo a dynamic change. PAGs are a family of aspartic proteinase-related glycoproteins expressed in the placental trophoblast cells of ruminants [26]; the expression levels of PAGs change according to the implantation status of embryos. Profiling hormonal changes and systemic metabolic perturbations at these discrete time points presents a unique opportunity to characterize the dynamic metabolic landscape underlying the opening, peak receptivity, and potential closure of the endometrial receptivity window.

Accordingly, this study investigates dynamic changes in the buffalo serum metabolome during key post-artificial insemination intervals (Days 15, 18, and 21). We systematically characterized metabolite profiles and pathway perturbations to identify metabolic signatures linked to embryonic implantation. Our specific aims were to characterize temporal fluctuations in the serum metabolome during the implantation window using high-throughput metabolomics and identify key metabolites and pathways demonstrating significant variation. Through these analyses, we elucidated the roles of these metabolites and pathways in regulating endometrial receptivity and mediating embryo–maternal crosstalk. This study may improve understanding of metabolic biomarkers for optimal receptivity. Ultimately, it could increase the conception rate in embryo transfer, thereby enhancing the reproductive efficiency of buffaloes.

## 2. Materials and Methods

### 2.1. Experiment Design

Healthy female water buffalo (4–10 years old, 1000–1500 kg, parity 1–5) used in this experiment were housed at the Breeding Farm of the Buffalo Research Institute in the Guangxi Zhuang Autonomous Region. All the animals were treated humanely as outlined in the Guide for the Care and Use of Experimental Animals of the National Institutes of Health, and the protocol was approved by the Animal Experiment Ethics Committee of Guangxi University (GXU-2024-106). Artificial insemination (AI) was performed within 14 to 16 h after the onset of estrus in female buffaloes, and 5 mL of fasting (14–16 h overnight) blood was collected from each of 22 buffaloes on days 15, 18, and 21 after AI for biochemical testing and serum metabolome sequencing. Fresh blood samples were left to rest for 45 min at room temperature and then centrifuged at 3000 rpm at 4 °C for 15 min to isolate serum. Serum samples were aliquoted into Eppendorf tubes and stored at −80 °C until further analysis. On day 40 after AI, pregnancy was checked using an ultrasound machine (HS-1600, Honda Electronics Co., Ltd., Toyota, Japan). The serum samples collected on days 15, 18, and 21 were categorized into two groups: the pregnant group (Preg: *n* = 12), which included samples from days 15, 18, and 21 post-AI, and the non-pregnant group (Non-P: *n* = 10), which also included samples from the same days.

### 2.2. Enzyme-Linked Immunosorbent Assay (ELISA)

Serum concentrations of E2, P4, and PAG were quantified using commercial enzyme-linked immunosorbent assay (ELISA) kits following the manufacturers’ instructions. All assays were performed in technical triplicate. The specific ELISA kits utilized were the E2 ELISA Kit (DLR-E2-Ge, Wuxi Donglin Technology Development Co., Ltd., Wuxi, China). The kit exhibits high specificity, with negligible cross-reactivity reported by the manufacturer for relevant steroids. The detection limit (LOD) was <0.062 ng/mL. P4 ELISA Kit (DLR-P4-Ge, Wuxi Donglin Technology Development Co., Ltd., Wuxi, China). The manufacturer reports high specificity. The LOD was <0.061 ng/mL. Bovine Pregnancy-Associated Glycoprotein (PAG) ELISA Kit (Catalog No. F0085-VA, Shanghai Kexing Trading Co., Ltd., Shanghai, China). The kit exhibits high specificity, with minimal cross-reactivity stated. The LOD was <1.0 ng/mL. Frozen serum samples were thawed at 4 °C and centrifuged at 3000× *g* for 15 min to remove particulates. Fifty microliters of standards and diluted serum samples were loaded into pre-coated wells. Except for the blank wells, 100 μL of horseradish peroxidase (HRP)-labeled detection antibody was added to each standard and sample well. The reaction wells were then sealed with sealing film and incubated at 37 °C in a water bath or incubator for 60 min. For washing, the liquid was discarded, and the wells were patted dry on absorbent paper. Each well was filled with washing solution and allowed to stand for 1 min. The washing solution was shaken off, and the wells were patted dry on absorbent paper. This washing step was repeated five times. Subsequently, 50 μL of substrate A and 50 μL of substrate B were added to each well and incubated at 37 °C for 15 min in the dark. Then, 50 μL of stop solution was added to each well. Plates were read within 10 min at 450 nm using a microplate reader (BioTek Epoch2; BioTek Instruments, Inc., Winooski, VT, USA).

### 2.3. Blood Cell Assays

Fresh whole blood samples were collected from healthy adult water buffaloes via jugular venipuncture. EDTA-K2 anticoagulant vacuum tubes (BD Biosciences, San Jose, CA, USA) with an anticoagulant concentration of 1.8 mg/mL were used. Samples were gently inverted 8–10 times immediately after collection to ensure homogeneous anticoagulant mixing and stored at 25 °C. All analyses were completed within 4 h post-collection to minimize cell degradation. On the instrument interface, select “Animal Mode”. Then, check “Buffalo” and set the analysis parameter to “CBC 5Diff RRBC”. The fully mixed blood collection tube was placed under the aspiration needle and injected for testing.

### 2.4. LC-MS Analysis

Serum samples were analyzed using the LC-MS system. Chromatographic separation parameters were based on prior studies [27,28]. Equal volumes from each serum sample were combined to prepare quality control (QC) samples. Ultra-high-performance liquid chromatography–mass spectrometry (UHPLC–MS) was performed with a Vanquish UHPLC system and an Orbitrap Q Exactive™ HF-X mass spectrometer (Thermo Fisher, Germering, Germany). The samples were introduced onto a Hypersil Gold column (100 × 2.1 mm, 1.9 µm) at a flow rate of 0.2 mL/min, employing a linear gradient over a duration of 17 min. For positive polarity, the eluent comprised eluent A (0.1% formic acid (FA) in water) and eluent B (methanol), while for negative polarity, eluent A consisted of 5 mM ammonium acetate at pH 9.0, paired with eluent B (methanol). The solvent gradient was as follows: 2% B for 1.5 min; increasing to 100% B over 12 min; held at 100% B until 14 min; decreasing to 2% B by 14.1 min; and returning to 2% B at 17 min [27,28].

### 2.5. Non-Targeted Metabolomic Analysis

Raw data files from UHPLC-MS/MS were analyzed using Compound Discoverer 3.1 (CD3.1, Thermo Fisher) in a non-targeted metabolomic framework. This workflow included selecting spectra, subtracting blank samples, identifying peaks, aligning retention times (RT), and comparing candidates with the mzCloud and ChemSpider databases. Additionally, mass spectrometry data were processed and evaluated using Compound Discoverer 3.0 (CD3.0) software. The analytical procedures were performed using the SIMCA-P 14.1 software suite (Umetrics, Umeå, Sweden), including principal component analysis (PCA) and orthogonal partial least squares discriminant analysis (OPLS-DA). Variable importance in projection (VIP) scores were derived from the OPLS-DA model. A VIP threshold > 1.2 and *p*-value < 0.05 were used to identify differential metabolites. Additionally, KEGG pathway analysis was performed using MetaboAnalyst 5.0, with a *p*-value < 0.05 indicating statistical significance.

### 2.6. Statistical Analysis

Statistical evaluations were conducted utilizing the SPSS 29.0 software (Chicago, IL, USA). Normality within the sample groups was assessed using the D’Agostino and Pearson test. Figures of PCA, OPLS-DA, and permutation tests were presented using the SIMCA-P 14.1 software suite (Umetrics, Umeå, Sweden). The volcano map and Venn diagram were plotted by the Hiplot online website (https://hiplot.com.cn/). Figures of the KEGG pathway were presented using MetaboAnalyst 5.0. Box plots and correlation heatmaps were presented using GraphPad Prism 8 software (San Diego, CA, USA). The T-test was used to compare the independent variables of the two groups. The results are expressed as means ± standard error of the mean (SEM). A *p*-value below or equal to 0.05 indicated statistical significance.

## 3. Results

### 3.1. Changes in Serum Hormones and Blood Leukocytes During the Buffalo Window of Implantation (WOI)

**Figure 1:**
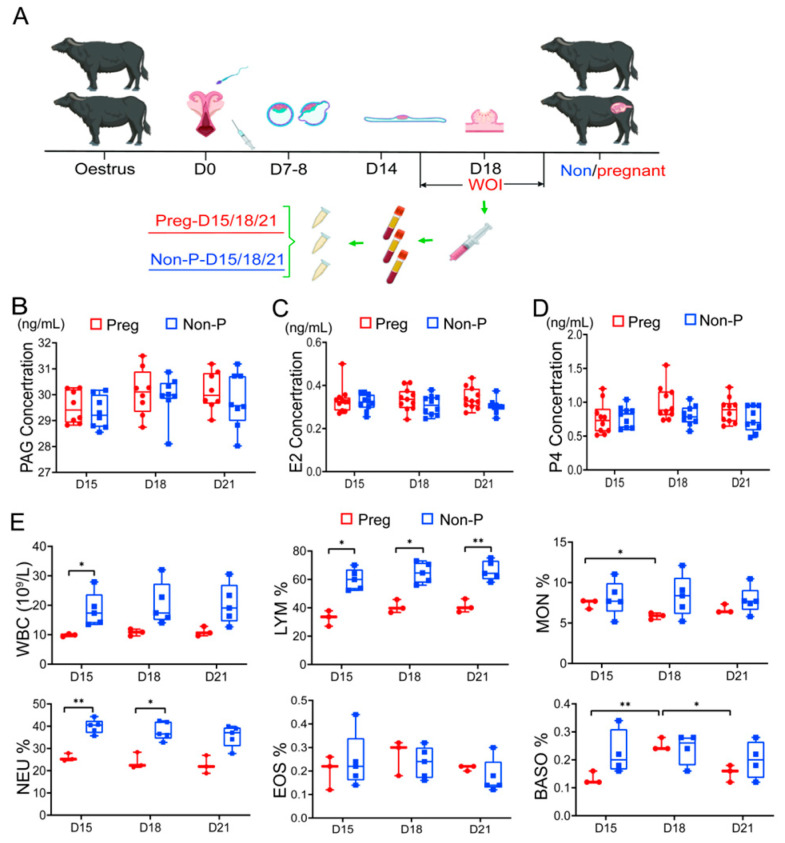
Changes in hormones and leukocytes during WOI of buffalo. (**A**) The animal experimental design. (**B**) The serum PAG levels during WOI of buffalo. (**C**) The serum E2 levels during WOI of buffalo. (**D**) The serum P4 levels during WOI of buffalo. (**E**) Changes in blood leukocytes during WOI of buffalo. Different labels (* and **) indicate significant differences (*p* < 0.05, *p* < 0.01), respectively.

Serum samples of buffaloes on days 15, 18, and 21 (D15/D18/D21) after artificial insemination were collected and divided into two groups based on pregnancy status: the pregnant group (Preg) and the non-pregnant (Non-P) group (Figure 1A). The concentrations of PAG, E2, and P4 in the serum were detected. Results showed that there was no significant difference in PAG concentration between the pregnant group and the non-pregnant group at all three time points (D15/D18/D21) (Figure 1B, Table S1). Although there were no significant differences in E2 and P4 concentrations between the Preg and Non-P groups, nor between the three time points (day 15/18/21) of each group, E2 and P4 in the Preg group showed a slow upward trend over time, while in the Non-P group, they showed a gradual downward trend (Figure 1C,D, Tables S2 and S3). Results indicated that the hormonal changes during the WOI were undetectable and can hardly be used as diagnostic markers of pregnancy. Further analysis of the total blood leukocyte count revealed that the white blood cell count (WBC), lymphocyte percentage (LYM%), and neutrophil percentage (NEU%) in the Preg group showed no significant differences among D15, D18, and D21 (Figure 1E, Table S4). However, the WBC in the Preg group was significantly lower than that in the Non-P group on D15 (*p* < 0.05), while the LYM% was significantly lower than that of the Non-P group (*p* < 0.05) on D15 and D18 and extremely significantly lower than that of the Non-P group (*p* < 0.01) on D21. Moreover, the NUE% was significantly lower than the Non-P group on D15 (*p* < 0.01) and D18 (*p* < 0.05) (Figure 1E, Table S4). In the pregnancy group, the percentage of basophils (BASO%) increased extremely significantly on D18 and then significantly decreased on D21 (Figure 1E, Table S4). The percentage of monocytes (MON%) decreased significantly on D18 and then slowly increased again on D21 (Figure 1E, Table S4). No significant differences were found in the percentages of eosinophils (EOS%) between the pregnant group and the Non-P group on D15, D18, or D21 (Figure 1E, Table S4). Data suggested that the embryo implantation process may be related to the immune microenvironment.

### 3.2. Changes in Serum Metabolites in Pregnant and Non-Pregnant Buffalo

**Figure 2:**
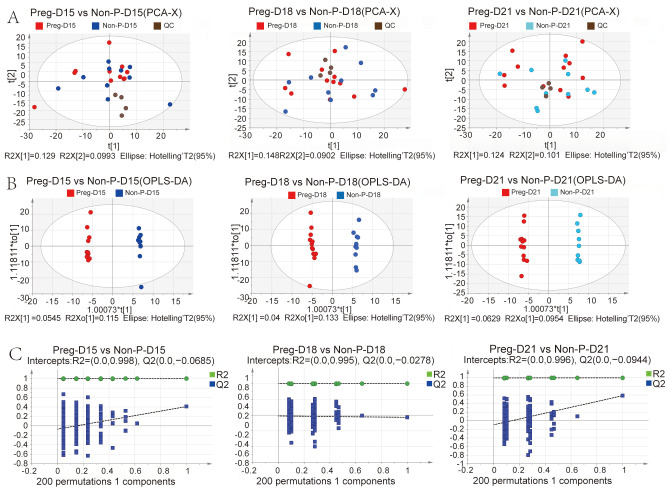
Quality control of metabolites. (**A**) The principal component analysis (PCA) of metabolites. (**B**) The OPLS-DA analysis of metabolites. (**C**) The OPLS-DA permutation test of metabolomics.

**Figure 3:**
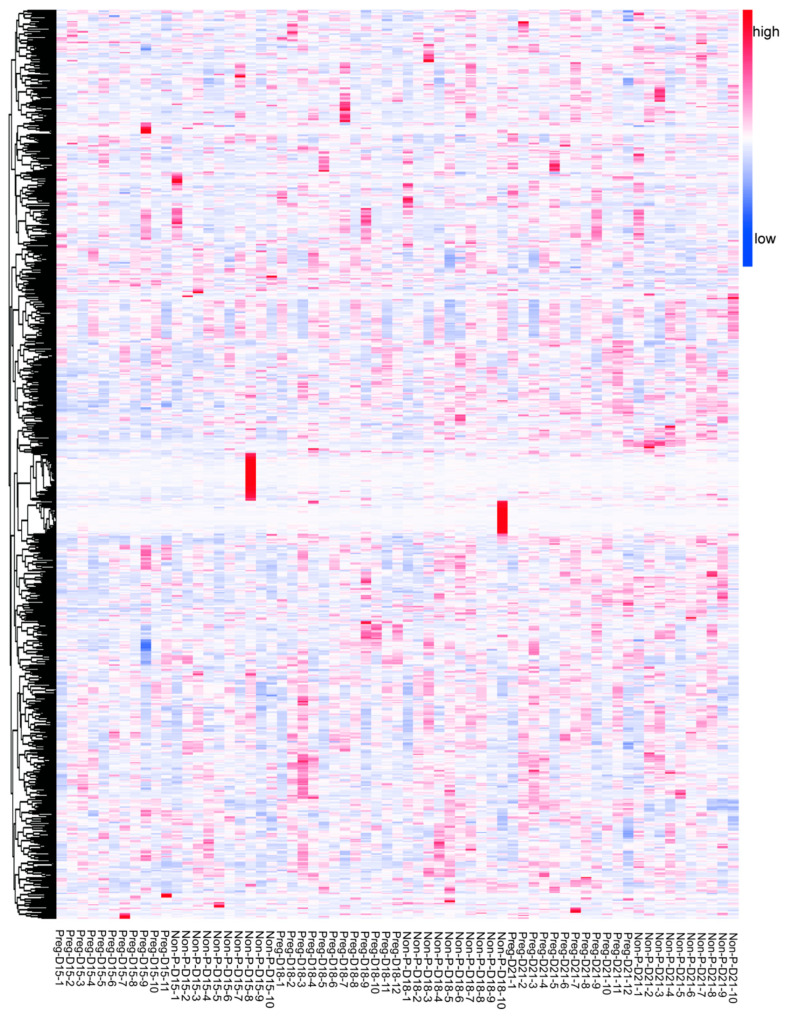
Total serum metabolites. Heatmap displaying the relative abundance profile of all 791 serum metabolites detected between the Preg and Non-P groups during D15-D18-D21. X-axis: Sample groups. Y-axis: Hierarchical clustering of serum metabolites (*n* = 791). Color scale: red indicates higher abundance relative to the mean; blue indicates lower abundance relative to the mean.

**Figure 4:**
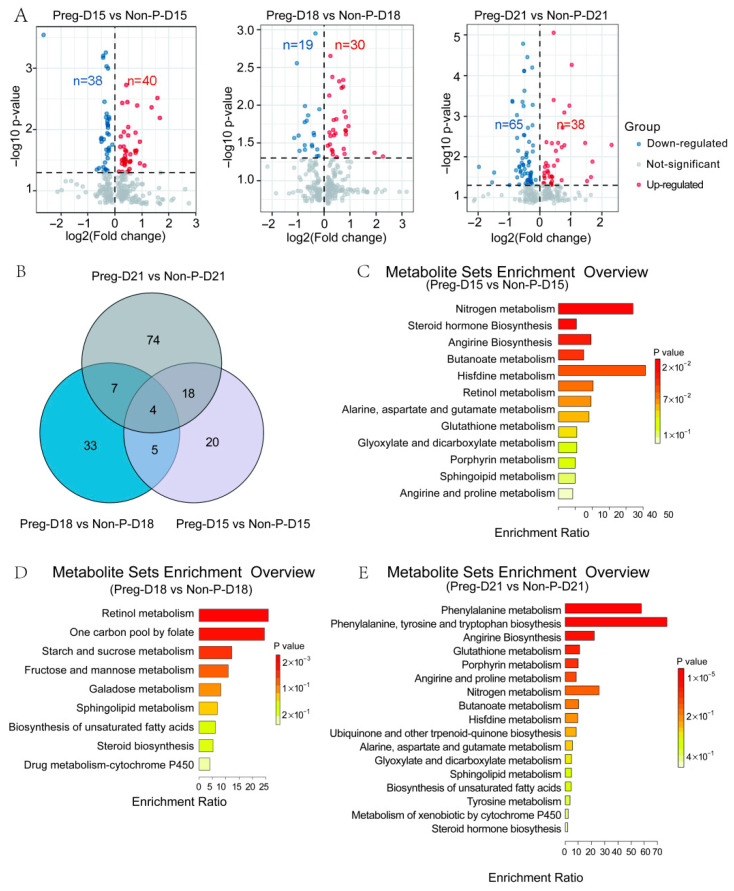
Comparison of serum metabolomic profiling between Preg and Non-P buffaloes. (**A**) Differential metabolites between pregnant and non-pregnant groups at different time points. Volcano plots visualizing the significance (*p*-value) versus magnitude (log_2_ fold change) of differential metabolites. Red dots represent significantly upregulated metabolites; blue dots represent significantly downregulated metabolites. (**B**) Venn diagram illustrating the overlap and uniqueness of significant differential metabolites identified at D15, D18, and D21. (**C**) KEGG pathway enrichment analysis of differential metabolites between Preg-D15 and Non-P-D15 groups. (**D**) KEGG pathway enrichment analysis of the differential metabolites between the Preg-D18 and Non-P-D18 groups. (**E**) KEGG pathway enrichment analysis of the differential metabolites between the Preg-D21 and Non-P-D21 groups.

Serum samples were collected from the Preg (*n* = 12) and Non-P (*n* = 10) groups on days 15, 18, and 21 post-insemination for metabolomic sequencing. One sample from the Preg group at day 15 was excluded due to collection failure, resulting in a final cohort of Preg (*n* = 11 on D15; *n* = 12 on D18/D21) and Non-P (*n* = 10 at each time point) for sequencing analysis. Principal component analysis (PCA) revealed distinct global metabolomic profiles between pregnant and non-pregnant groups at each gestational day (Figure 2A). Two principal components (PC1 and PC2), which captured the largest sources of variation in the dataset, predominantly reflected the pregnancy status as evidenced by the clear separation between groups within each time point. Specifically, the variance explained by PC1 and PC2 was as follows: Day 15: PC1 = 12.9% (R2X[1] = 0.129), PC2 = 9.93% (R2X[2] = 0.0993); Day 18: PC1 = 14.8% (R2X[1] = 0.148), PC2 = 9.02% (R2X[2] = 0.0902); and Day 21: PC1 = 12.4% (R2X[1] = 0.124), PC2 = 10.1% (R2X[2] = 0.101). Visualization of the samples projected onto the plane defined by PC1 and PC2 is presented in Figure 2A. Critically, the 95% confidence ellipses (based on Hotelling’s T^2^ statistics, α = 0.05) surrounding the group centroids showed minimal overlap and clear separation between the Preg and Non-P groups at all three time points. This statistically significant group separation (*p* < 0.05 via Hotelling’s T^2^ test) indicates that the global metabolic compositions differed significantly between pregnancy states throughout the examined time course. Orthogonal partial least squares discriminant analysis (OPLS-DA) showed significant separation between the groups (Figure 2B). The original R2 and Q2 values all exceed the permuted counterparts during a 200-iteration permutation test, which validated the reliability of the analysis model (Figure 2C). A total of 791 serum metabolites were detected across all samples. The overall number of metabolites and the distinct patterns between the Preg and Non-P groups are visually summarized in Figure 3 (see Table S5 for the full dataset). Subsequent analysis highlighted significant differences in metabolic abundance profiles between the groups. Comparative analysis identified distinct patterns of metabolic changes across gestational timepoints. Differential metabolites were identified and visualized using volcano plots and assessed for overlap using a Venn diagram (Figure 4A,B). On D15, 47 differentially expressed metabolites (DEMs) were identified between the pregnant (Preg-D15) and non-pregnant (Non-P-D15) groups, with 20 significantly upregulated and 27 significantly downregulated (Figure 4A). On D18, 49 DEMs were found between pregnant (Preg) and non-pregnant (Non-P) samples, consisting of 30 upregulated and 19 downregulated metabolites (Figure 4A). Notably, the highest number of DEMs (103) was found on D21, comprising 38 upregulated and 65 downregulated metabolites (Figure 4A). The Venn diagram in Figure 4B illustrates that while each time point exhibited a substantial number of unique DEMs, a core set of DEMs was commonly shared across all three stages. Further KEGG enrichment analysis showed that DEMs between Preg-D15 and Non-P-D15 were mainly enriched in pathways such as nitrogen metabolism, steroid hormone biosynthesis, and arginine biosynthesis (Figure 4C). The DEMs between Preg-D18 and Non-P-D18 were mainly enriched in pathways such as retinol metabolism, one-carbon pool by folate, and starch/sucrose metabolism (Figure 4D). While for the data of D21, DEMs were mainly enriched in pathways related to phenylalanine metabolism, phenylalanine/tyrosine/tryptophan biosynthesis, and arginine biosynthesis (Figure 4E). Results revealed stage-specific pathway activation: steroid hormone biosynthesis on D15 establishes a receptive endometrial environment for implantation, while retinol metabolism on D18 provides essential substrates for subsequent organogenesis. By D21, significant perturbations in aromatic amino acid pathways suggest a preparatory maternal metabolic shift that anticipates the need to allocate essential nutrients to support the rapid growth of the conceptus following implantation.

### 3.3. Changes in Serum Metabolites During the Buffalo WOI

**Figure 5:**
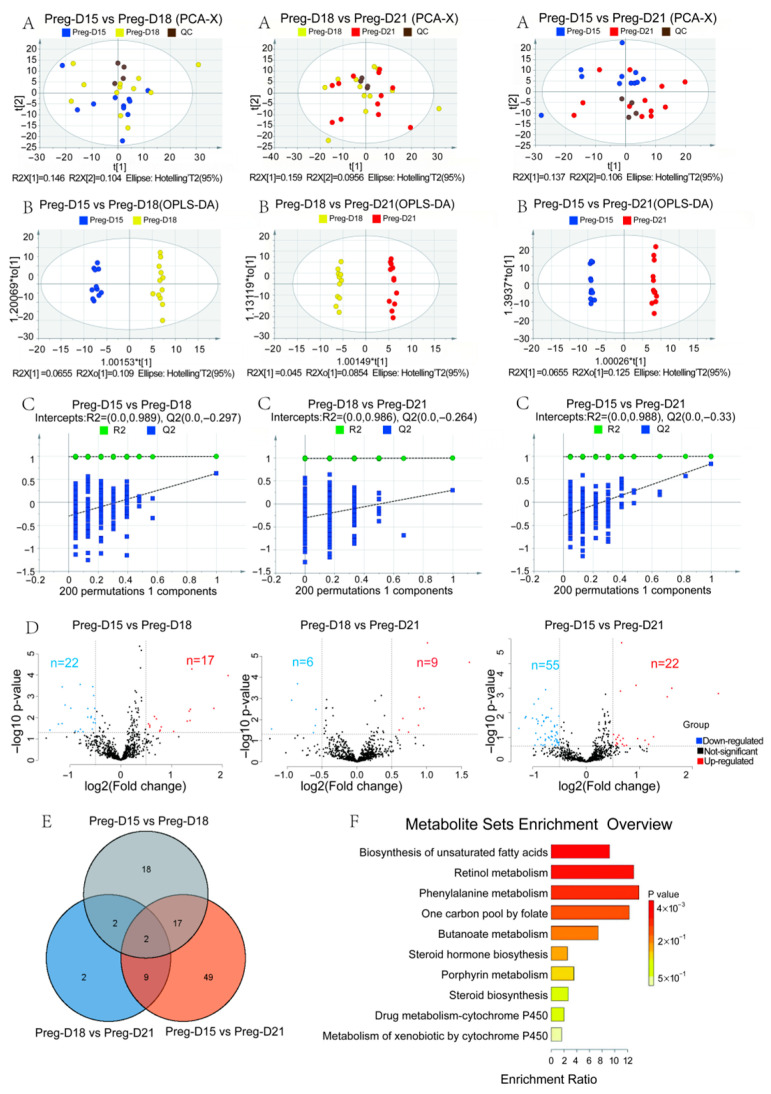
Changes in serum metabolites during WOI of buffalo. (**A**) The principal component analysis (PCA) of metabolites. (**B**) The OPLS-DA analysis of metabolites. (**C**) The OPLS-DA permutation test of metabolomics. (**D**) The identification of differential metabolites. (**E**) A Venn diagram showing differential metabolites. (**F**) The KEGG pathway enrichment analysis of differential metabolites.

To further understand the differences in serum metabolites of buffaloes pre-implantation, peri-implantation, and early post-attachment, the serum metabolome of pregnant buffaloes on D15, D18, and D21 was analyzed. PCA showed that the variation between D15, D18, and D21 was all below 16% (Figure 5A). While OPLS-DA analysis showed significant separation between groups (Figure 5B). The permutation test showed the models were reliable (Figure 5C). In this study, 39 DEMs were identified between the D15 and D18 groups, including 17 upregulated and 22 downregulated metabolites (Figure 5D). Between the D18 and D21 groups, nine upregulated and seven downregulated DEMs were identified. Moreover, 77 DEMs were identified between the D15 and D21 groups, including 22 upregulated and 55 downregulated metabolites (Figure 5D). Two common DEMs (N′1-[1-(2-hydroxyphenyl)ethylidene]-3-methoxybenzene-1-carbohydrazide and LNAPE 20:4/N-16:0) were identified between the groups (Figure 5E), while quercetin-3β-D-glucoside, LNAPE 18:2/N-22:5, Valdecoxib, bilirubin, and 5-methyltetrahydrofolic acid showed significant differences between D15 and D18, and quercetin-3β-D-glucoside, as a flavonoid glycoside, had the same antioxidant and anti-inflammatory properties, which could significantly improve embryo attachment by enhancing uterine receptivity. Valdecoxib’s anti-inflammatory effects inhibit excessive inflammatory responses early in implantation (D15), thereby promoting embryo implantation. Bilirubin, as a bile acid metabolite, may affect the uterine environment by modulating inflammation and oxidative stress. 5-Methyltetrahydrofolic acid is involved in folate metabolism, which is associated with one-carbon unit transfer and DNA synthesis, affecting embryonic development. These metabolites collectively influenced pregnancy outcomes in buffalo from pre-implantation to implantation. KEGG analysis showed that the 77 DEMs were mainly enriched in 10 KEGG pathways, including biosynthesis of unsaturated fatty acids, retinol metabolism, and phenylalanine metabolism (Figure 5F). Together, the enrichment of these KEGG pathways suggests that buffalo metabolic reprogramming during implantation focuses on three core functions: immunomodulation (e.g., anti-inflammatory and antioxidant), cell differentiation support (e.g., embryonic and placental development), and energy/substrate supply optimization.

### 3.4. Correlation Analysis of Metabolomics and Leukocytes

**Figure 6:**
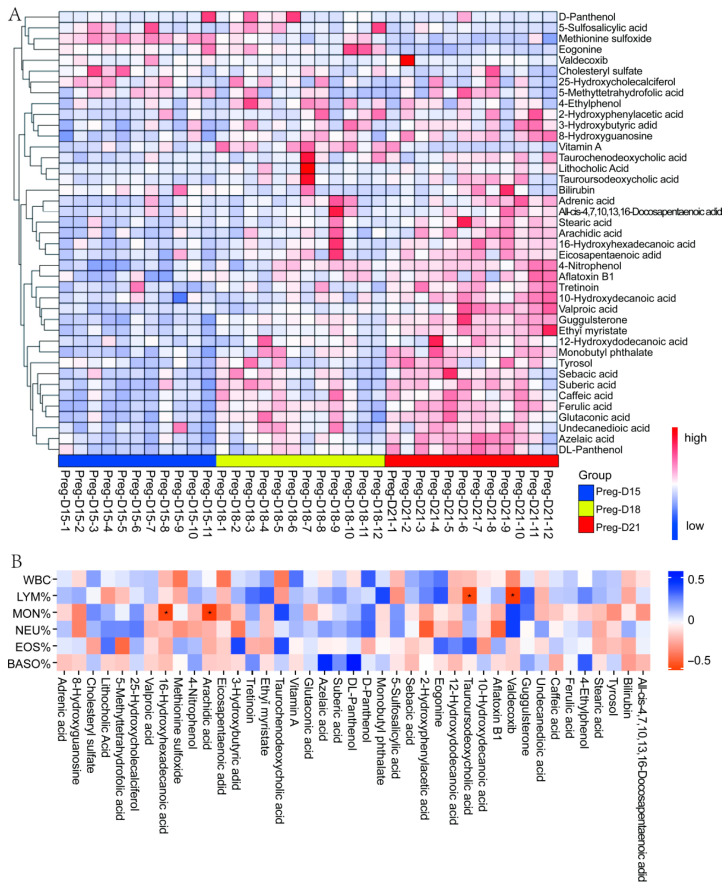
Correlation analysis of DEMs and blood leukocytes. (**A**) Cluster analysis of DEMs. (**B**) Correlation analysis of shared DEMs and blood leukocytes. Different labels (*) indicate significant differences (*p* < 0.05).

Cluster analysis indicated that the DEMs were clustered separately at the different periods (D15, D18, D21) of the embryo implantation window (Figure 6A). Correlation analysis showed that 16-hydroxyhexadecanoic acid and arachidic acid were significantly negatively correlated with the percentage of monocytes (MON%), while tauroursodeoxycholic acid and Valdecoxib were significantly negatively correlated with the percentage of lymphocytes (LYM%). Valdecoxib, bilirubin, D-panthenol, lithocholic acid, and 25-hydroxycholecalciferol were positively correlated with the percentage of neutrophils (NEU%), but the correlations were not significant. Vitamin A, D-panthenol, and Ecgonine were positively correlated with the number of white blood cells (WBCs), but the correlations were not significant. (Figure 6B). Results suggest that during the buffalo embryo implantation window, 16-hydroxyhexadecanoic acid, arachidic acid, tauroursodeoxycholic acid, and Valdecoxib may promote immune tolerance and reduce immune attack, which is beneficial for pregnancy. The non-significant positively correlated metabolites (such as vitamin A, bilirubin, or 25-hydroxycholecalciferol) may be involved in maintaining immune homeostasis or mild inflammatory regulation.

## 4. Discussion

In studies investigating serum hormone dynamics during early pregnancy in buffaloes, the regulatory patterns of PAGs, E2, P4, and immune cell parameters exhibit distinct biological characteristics. As a hallmark protein secreted by ruminant placental trophoblasts, the functional role of PAGs in early buffalo pregnancy remains contentious. However, elucidating these complex interactions is crucial for developing refined reproductive strategies. Specifically, defining the normative profiles of these biomarkers can provide a foundation for reliable early pregnancy diagnosis, improve the ability to identify pregnancies at risk of failure, and ultimately enhance overall herd reproductive management and genetic selection efficiency in buffalo production systems. Compared to cattle, buffalo PAG secretion appears more tightly linked to placental developmental stages, implying that integrating ultrasound-based placental morphometry could enhance detection precision. Research has found that buffalo placentas can specifically express PAG subtypes [29]. However, early serum assays reveal no significant disparity between pregnant and non-pregnant, which is consistent with the present study. In addition, Munna et al. [25] successfully detected significant expression of PAG on day 42 after pregnancy, while no reliable difference was observed at earlier time points (e.g., days 0 or 30). This may be due to the complex post-translational modifications of buffalo PAGs and the incomplete match of the detection windows with the secretion peaks. The synergistic regulation of E2 and P4 is pivotal for maintaining buffalo pregnancy. Serum E2 and P4 levels in pregnant groups display a gradual upward trajectory, whereas non-pregnant groups exhibit a progressive decline, which is consistent with the observations in Egyptian buffalo, where E2 peaks in the 10th month of gestation. The incremental elevation of E2 may participate in regulating placental angiogenesis and endometrial receptivity, though specific molecular pathways (e.g., estrogen receptor signaling) require deeper elucidation [30]. Additionally, the immune microenvironment of pregnant buffaloes features distinctive traits: total WBC, LYM%, and NEU% are extremely significantly lower than in non-pregnant groups, mirroring the enhanced Th2-type immune response mechanism observed in pregnant African buffaloes. BASO% transiently increases on D18 before declining, potentially related to local mast cell activation during decidualization. In stillbirth cases, elevated pro-inflammatory cytokines such as IL-6 and IFN-γ suggest immune dysregulation may precipitate pregnancy failure [31,32,33]. This immunosuppressive state presumably arises from modulated maternal tolerance to fetal antigens, though the roles of specific regulators (e.g., IDO enzyme, Treg cells) remain unclear. Current research highlights species-specificity in early pregnancy hormonal regulation and immune adaptation in buffaloes. Nevertheless, PAG detection methodologies require optimization, and integrating single-cell sequencing to characterize dynamic changes in immune cell subsets during pregnancy is imperative. Such investigations will provide theoretical support for improving buffalo reproductive efficiency and reducing embryo loss.

Analysis of serum metabolites in buffaloes at different early pregnancy and non-pregnancy time points identifies stage-specific enrichment of DEMs in metabolic pathways, which may influence pregnancy outcomes by regulating maternal metabolic adaptation, embryonic development, and maternal–fetal crosstalk. On D15, DEMs between pregnant and non-pregnant groups primarily enrich nitrogen metabolism, steroid hormone biosynthesis, and arginine biosynthesis pathways. Our results identified a significant enrichment of DEMs associated with nitrogen metabolism pathways in pregnant buffaloes at D15, including the upregulation of key metabolites such as L-glutamic acid, methionine sulfoxide, and D-sphingosine. The elevation of these specific metabolites strongly supports the role of this pathway in supplying amino acid substrates necessary for embryonic protein synthesis, thereby helping to ensure a nutritive microenvironment conducive to blastocyst development. Steroid hormone biosynthesis regulates P4 and E2 production to establish endometrial receptivity, laying the foundation for implantation; notably, arginine, a key pathway product, promotes endometrial vasodilation and neovascularization via nitric oxide (NO) generation, a process likely crucial for enhancing maternal–fetal material exchange efficiency and facilitating successful implantation. In our study, metabolomic analysis revealed distinct temporal patterns of differentially expressed metabolites (DEMs) in the serum. Specifically, on D18, we observed that the DEMs were significantly enriched in pathways related to retinol metabolism, folate-mediated one-carbon pool, and starch/sucrose metabolism. The following enrichment pattern suggests potential biological roles in preparing the endometrium for embryo arrival: Retinol (vitamin A) metabolism: Changes in DEMs within this pathway may indicate altered metabolic flux toward retinoic acid, a morphogen known to regulate embryonic cell differentiation and morphogenesis [34,35], processes vital for ensuring orderly germ layer formation prior to implantation. Folate-mediated one-carbon pool: The enrichment of DEMs here points to potential modulations in folate-dependent processes, which are critical for DNA methylation and nucleotide synthesis [36]. These activities are essential for maintaining genomic stability during the rapid cell proliferation characterizing the pre-implantation embryo and could help mitigate the risk of aneuploidy. Starch/Sucrose metabolism: DEMs associated with this pathway likely reflect adaptations to meet the surging energy demands of the transitioning blastocyst. Specifically, alterations in DEMs involved in carbohydrate breakdown might facilitate increased glucose availability, serving as a primary energy substrate for the developing embryo as it prepares for gastrulation. Subsequently, on D21, our DEM profile shifted significantly, with enrichment concentrated in pathways of phenylalanine metabolism, phenylalanine/tyrosine/tryptophan biosynthesis, and arginine biosynthesis. This transition in pathway enrichment suggests a focus shift toward supporting the establishment of the maternal–fetal interface post-attachment. Phenylalanine metabolism and phenylalanine/tyrosine/tryptophan biosynthesis: The prominent enrichment of DEMs in these aromatic amino acid pathways implies potential roles beyond mere nutrient provision. We speculate that the observed metabolic changes could support the synthesis of neurotransmitters (e.g., dopamine and serotonin) derived from these precursors [37,38] and/or contribute to immune tolerance mechanisms. Notably, metabolites of tryptophan, such as kynurenine, are known inducers of indoleamine 2,3-dioxygenase (IDO) expression. IDO activity creates a local immunosuppressive environment by inhibiting maternal T-cell responses to fetal antigens, thereby facilitating the establishment of critical maternal–fetal immune tolerance during the crucial early post-implantation phase [39,40]. Arginine biosynthesis: The enrichment of DEMs related to arginine biosynthesis on D21 further underscores the potential importance of this amino acid beyond its role mentioned earlier. Elevated arginine availability via enhanced biosynthesis could synergistically promote sustained nitric oxide (NO) production, supporting the ongoing neovascularization and vasodilation needed for efficient placental development and nutrient/waste exchange after implantation is initiated. The recurrent enrichment of arginine biosynthesis underscores its sustained role in placental vascular network expansion—enlarging embryos on D21 require more robust circulatory systems for nutrient exchange. Collectively, these stage-specific pathways synergistically regulate pregnancy outcomes: D15 pathways focus on implantation preparation, D18 pathways prioritize maintaining developmental stability, and D21 pathways emphasize immune tolerance and nutrient supply. Dysregulation of these pathways in non-pregnant groups may cause hormonal insufficiency, energy depletion, or immune rejection, ultimately leading to pregnancy failure.

Further analysis of metabolic dynamics across D15, D18, and D21 in pregnant buffaloes reveals associations with endometrial receptivity during the implantation window. PCA of the serum metabolome revealed limited overall variation (<16%) among samples from days 15, 18, and 21 of pregnancy. This pattern of overlapping clusters with only subtle separation on early components indicates a gradual transition in the metabolic profile across these critical time points. Although this overall pattern of gradual transition is suggested by PCA, we can discern more subtle differences through supervised multivariate analysis. Orthogonal Partial Least Squares-Discriminant Analysis (OPLS-DA) models constructed between pairwise time points (D15–D18, D18–D21, and D15–D21) demonstrated a clear group separation. This distinct separation validates the existence of unique metabolic signatures corresponding to key stages: receptivity establishment (D15–D18), receptivity maintenance (D18–D21), and comprehensive remodeling (D15–D21). Thirty-nine DEMs between D15 and D18 indicate a metabolically active phase transitioning from non-receptive to receptive endometrium. KEGG pathway enrichment analysis of the DEMs distinguishing D15 from D18 highlighted retinol metabolism as one of the significantly altered pathways. It is plausible that these shifts regulate endometrial epithelial cell differentiation dynamics in preparation for receptivity, potentially influencing the expression of critical adhesion molecules such as integrins, thereby facilitating successful embryo attachment. We found only 15 DEMs between D18 and D21, which indicates metabolic stabilization during the stage of receptivity maintenance; activated unsaturated fatty acid biosynthesis may enhance membrane fluidity to support trophoblast–endometrial adhesive interactions, sustaining the receptive window. Seventy-seven DEMs between D15 and D21 (including phenylalanine metabolism) reflect comprehensive metabolic remodeling from receptivity initiation to maintenance. Phenylalanine metabolism may regulate maternal–fetal immune tolerance via tryptophan catabolism (e.g., indoleamine 2,3-dioxygenase-mediated pathways), preventing endometrial immune rejection and ensuring sustained receptivity. Only two shared DEMs highlight stage-specific metabolic traits, aligning with the spatiotemporal specificity of the “transient” endometrial receptivity window. These metabolic changes synergistically support receptivity establishment (D15-D18) and maintenance (D18-D21) through energy provision, cellular signaling, and immune microenvironment balance, providing critical metabolic foundations for successful embryo implantation.

Additionally, preliminary analysis of blood leukocyte dynamics and pregnancy immune tolerance during the buffalo WOI identifies significantly increased regulatory T cells (Treg) in WOI blood, which secrete IL-10 and TGF-β to suppress Th1-type immunity, preventing endometrial embryo rejection. Concurrently, dominant Th2-type responses promote trophoblast invasion and placental angiogenesis [41]. This balance may involve pregnancy-associated glycoproteins (PAGs), as buffalo placental PAGs induce endometrial cells to secrete neutrophil chemokines, regulating leukocyte migration and inhibiting excessive inflammation [42,43]. Furthermore, increased blood M2 macrophage proportions create an immunosuppressive implantation microenvironment by secreting anti-inflammatory factors (e.g., IL-10) and promoting angiogenesis. Studies indicate embryos achieve immune evasion via HLA-G expression; trophoblast-specific HLA-G binds maternal NK cell inhibitory receptors (e.g., KIR2DL4), blocking perforin/granzyme-mediated cytotoxicity to avoid “foreign” recognition [44,45]. Reduced NK cell activity in buffalo WOI blood may relate to this mechanism. In this study, we identified spatiotemporal specificity in WOI leukocyte distribution, e.g., transient increases in neutrophils during D15-D18 may clear pathogens but require PAG-mediated pathway regulation to prevent embryonic damage; trophoblast cells sustain high immunomodulatory molecule expression during D18-D21, inhibiting effector T cell function via cell-contact mechanisms to maintain tolerance. As seasonal breeders, buffaloes may exhibit unique WOI immune regulation—embryos likely utilize PAG-induced neutrophil chemotaxis to maintain local immune homeostasis rather than classical HLA-G pathways. However, the immune evasion molecules of buffalo trophoblasts and PAG–leukocyte interaction mechanisms warrant further investigation.

While this metabolomic analysis identifies novel associations between serum metabolites and endometrial receptivity during the implantation window in buffalo, the following limitations warrant consideration. First, heterogeneity in maternal age and parity within the cohort introduces potential confounding variables, as reproductive experience and age are known modulators of endometrial function and steroid responsiveness. Second, while statistically powered for primary pathway analysis, the sample size may constrain detection of subtle metabolite–immune interactions or rare biomarkers, limiting granular subgroup analyses. Third, sampling is limited to a short period of time during the implantation window (days 15–21 after insemination). Given the dynamic nature of embryo–maternal crosstalk, increasing sampling time points (specifically pre-implantation (e.g., day 7) and post-attachment (e.g., after day 21) for metabolic analysis would better describe temporal immunometabolic changes, although resource constraints preclude this design. Future studies should prioritize longitudinal sampling in standardized cohorts and correlate metabolic signatures with direct measures of pregnancy success to validate these mechanisms.

## 5. Conclusions

This study systemically analyzed the relationship between serum metabolite changes and endometrial receptivity during the embryo implantation window in buffaloes using metabolomics. We found that the metabolic profile associated with implantation is potentially linked to the types of immune cells and metabolites in the blood. The DEMs were mainly related to steroid, retinol, one-carbon, fatty acid, and phenylalanine metabolism, suggesting that immune metabolism may play essential roles in modulating endometrial receptivity during the implantation window. This study provides potential clues and a metabolic framework for understanding the mechanisms underlying buffalo embryo implantation. These findings lay the groundwork for future research aimed at improving the reproductive efficiency of buffalo, which should include direct measures of gestation success to validate these associations.

## Data Availability

All datasets used and/or analyzed during the current study are available from the corresponding author upon reasonable request.

## Supplementary Materials

The following supporting information can be downloaded at: https://www.mdpi.com/article/10.3390/metabo15090615/s1, Table S1: Serum PAG levels; Table S2: Serum E2 levels; Table S3: Serum P4 levels; Table S4: Blood leukocyte count; Table S5: Serum metabolites.

## Abbreviations

The following abbreviations are used in this manuscript:

WOI	Window of Implantation
D15	D15 of artificial insemination
D18	D18 of artificial insemination
D21	D21 of artificial insemination
Preg	pregnant
Non-P	non-pregnant
